# STAT3 Regulates Mouse Neural Progenitor Proliferation and Differentiation by Promoting Mitochondrial Metabolism

**DOI:** 10.3389/fcell.2020.00362

**Published:** 2020-05-19

**Authors:** Yixun Su, Wenjun Zhang, C. Pawan K. Patro, Jing Zhao, Tianhao Mu, Zhongnan Ma, Jianqiang Xu, Kenneth Ban, Chenju Yi, Yi Zhou

**Affiliations:** ^1^The Seventh Affiliated Hospital, Sun Yat-sen University, Shenzhen, China; ^2^Department of Biochemistry, Yong Loo Lin School of Medicine, National University of Singapore, Singapore, Singapore; ^3^Neurobiology Programme, Life Sciences Institute, National University of Singapore, Singapore, Singapore; ^4^School of Medicine, Indiana University, Indianapolis, IN, United States; ^5^Cancer Science Institute of Singapore, Singapore, Singapore; ^6^Department of Biology, Southern University of Science and Technology, Shenzhen, China; ^7^West China Hospital, Sichuan University, Chengdu, China; ^8^Model Animal Research Center of Nanjing University, Nanjing, China

**Keywords:** neural progenitor, neurogenesis, proliferation, STAT3, mitochondria, OXPHOS

## Abstract

The proliferation and differentiation of neural progenitor lay the foundation for brain development. In neural progenitors, activation of Signal Transducer and Activator of Transcription 3 (STAT3) has been found to promote proliferation and astrocytogenesis while suppressing neurogenesis. However, our study found that *Stat3* conditional knockout in neural progenitors (*Stat3* cKO) also results in increased proliferation and suppressed neurogenesis. To investigate how STAT3 regulates these processes, we attempted to identify potential STAT3 target genes by RNA-seq profiling of the control (CTL) and *Stat3* cKO neural progenitors. We found that STAT3 promotes the expression of genes involved in the mitochondrial oxidative phosphorylation (OXPHOS), and thereby promotes mitochondrial respiration and negatively regulates reactive oxygen species (ROS) production. In addition, we demonstrated that *Stat3* loss-of-function promotes proliferation via regulation of mitochondrial metabolism and downstream signaling pathways. Our study provides novel insights into the relation between STAT3, mitochondrial metabolism and the process of embryonic neurogenesis.

## Introduction

Neural development is a complex, orchestrated process consists of multiple elements, including the self-renewal and differentiation of neural progenitors. Neural progenitors divide to self-renew and give rise to neurons in the early embryonic stage, and they become gliogenic in the later stages (Bayer and Altman, 1991; Kriegstein and Alvarez-Buylla, 2009). The self-renewal and differentiation of neural progenitors are fine-tuned by transcriptional factors and other pathways (Martynoga et al., 2012). Dysregulation of these processes could lead to severe developmental defects such as microcephaly and lissencephaly (reduced neurogenesis), or megalocephaly and autism spectrum disorder (overproduction of neurons) (Mochida, 2009; Fang et al., 2014).

Signal transducer and activator of transcription 3 (STAT3) plays an important role in neural progenitor self-renewal and differentiation. As a member of the STAT family, STAT3 responds to cytokines and growth factors, and is then phosphorylated at tyrosine 705 residue by gp130/LIFR-associated JAK kinase, translocates into the nucleus and activates transcription of its target genes (Fu et al., 1990, 1992; Schindler et al., 1992; Darnell et al., 1994; Levy and Darnell, 2002). In addition, STAT3 also promotes gene expression in a tyrosine-phosphorylation-independent manner (Yang et al., 2005, 2007).

In embryonic neural progenitors, STAT3 has been found to be sufficient and necessary for astrocytogenesis: activation of STAT3 by IL6 family cytokines promotes the astrocyte marker GFAP expression, which is diminished in the Stat3 conditional knockout (Bonni et al., 1997; Nakashima et al., 1999; Hong and Song, 2014). Mechanistically, activated STAT3 form a complex with SMAD1 and p300 and bind to the GFAP promoter to induce its expression (Nakashima et al., 1999). During the neurogenesis phase, activation of STAT3 also promotes cell proliferation and negatively regulates neurogenesis (Gu et al., 2005; Yoshimatsu et al., 2006; Cao et al., 2010; Chen et al., 2013; Hong and Song, 2015). However, knockout of STAT3 in astrocytes also resulted in a decreased proliferation rate (de la Iglesia et al., 2008).

STAT3 has been shown to be involved in mitochondrial metabolism in the central nervous system, which might play an important role in cell survival and axon outgrowth (Guo et al., 2008; Sarafian et al., 2010; Luo et al., 2016), but the causal mechanism remained unclear. It has also been shown that STAT3 could be translocated into mitochondria, and then promotes respiration by binding to protein complexes such as Complex I, or by regulating mitochondrial transcription (Wegrzyn et al., 2009; Macias et al., 2014; Carbognin et al., 2016; Xu et al., 2016). However, whether mitochondrial STAT3 could regulate mitochondrial metabolism was still controversial (Phillips et al., 2010). Furthermore, our previous report found that STAT3 does not localize in mitochondria, but in the mitochondria associated membranes (Su et al., 2019). Thus, STAT3 might regulate mitochondrial metabolism via other pathways.

In this study, we found that conditional knockout of STAT3 in neural progenitors also leads to increased proliferation and deteriorated neurogenesis. In addition, we found that STAT3 is not necessary for astrocyte differentiation, but instead only required for the expression of GFAP. To investigate the role of STAT3 in neural progenitor, we used RNA-seq analysis to uncover STAT3-dependent genes. We demonstrated that STAT3 promotes expression of genes that involve in the oxidative phosphorylation pathway and thereby promotes mitochondrial respiration. We demonstrated that this regulation is critical for *Stat3*-mediated neurogenesis. Our findings provide novel insights into the link between mitochondrial metabolism and the process of neurogenesis in brain development.

## Materials and Methods

### Animals

The generation of mice with a conditional KO of *Stat3* has been described (Welte et al., 2003; Moh et al., 2007). Exons 18–20, which contain the SH2 domain of STAT3, were flanked by two lox P sites. Nestin-Cre transgenic mice [The Jackson Laboratory mice database: B6.Cg(SJL)-TgN(Nes-cre)1Kln] expressing Cre under the control of a rat Nestin promoter/enhancer were described (Tronche et al., 1999). The Nestin-Cre *Stat3-flox/flox* (*Stat3-f/f*) mutant mice, designated as *Stat3* cKO, were generated by mating Nestin-Cre *Stat3-f/*+ mice with *Stat3-f/f* mice. The genotype was determined by PCR as described (Welte et al., 2003). The genotyping primers sequence is listed in Supplementary Table S1. The sample result of genotyping is shown in Supplementary Figure S1. As the transgenic Nestin-Cre expression has been reported to cause metabolic changes (Harno et al., 2013), here we use Nestin-cre; *Stat3*-flox/+ embryos as control (C; F/+) to account for the possible confounding effects. In addition, the control (C; F/+) mice are born in Mendelian ratios without observable growth defects. All experiments were done in a C57BL6 background. All procedures were performed in accordance with the National Institutes of Health Guide for the Care and Use of Laboratory Animals and under the approval of the NUS Institutional Animal Care and Use Committee.

### The Cell Culture of Primary Embryonic Neural Progenitors

The primary neural progenitor culture was achieved according to an established protocol (Pacey et al., 2006) with some adjustment. In brief, pregnant mice were sacrificed by CO_2_ overdose at the designated pregnancy stage. Embryonic brains were dissected and subjected to digestion with Accutase (Thermo Fisher Scientific) to isolate neural progenitors. The cells were then subjected to suspension culture in serum-free medium (SFM) supplemented with EGF and FGF at the density of 2 × 10^5^ cells/ml. Fresh medium was replenished every other day. Cells grown into neurospheres were collected on the 3rd day for RNA/protein sample preparation. For differentiation assay in attachment culture, neurospheres were dissociated and cells were seeded onto poly-D-lysine and laminin (Sigma-Aldrich) coated coverslip at the density of 1 × 10^5^ cells/ml in SFM with EGF/FGF (Sigma-Aldrich) supplement. To differentiate neural progenitors into neurons and glial cells, EGF and FGF were withdrawn on the 1st day after attachment. Half of the SFM was changed every other day, and the cells were collected for immunostaining or RNA/protein sample preparation. The detailed components of SFM and their source can be found in Supplementary Table S2.

### RNA-seq

Neural progenitors isolated at E11 and E14 wild-type or *Stat3* cKO embryos were collected after culture for 3 days and were lysed directly by Trizol (Thermo Fisher Scientific) and total RNA was isolated according to manufacturer’s protocol. cDNA was obtained using the M-MLV Reverse Transcriptase kit from Promega according to the manufacturer’s protocol. RNA-seq experiment was carried out using Illumina Hiseq 2000 Sequencing System. The sequence files obtained were then undergone a quality check by FastQC, then processed and analyzed using the Tuxedo pipeline (Trapnell et al., 2012). Differentially expressed genes were then clustered using Database for Annotation, Visualization and Integrated Discovery (DAVID) analysis (Huang da et al., 2009a, b) for data mining.

### RNA-Extraction and RT-qPCR

Neural progenitors were lysed directly by RNAzol (Sigma-Aldrich) and total RNA was isolated according to the manufacturer’s protocol. Reverse transcription was carried out with the M-MLV Reverse Transcriptase kit from Promega according to the manufacturer’s protocol. qPCR experiment was carried out using SYBR green qPCR kit from KAPA and Applied Biosystems 7500 Real PCR System. qPCR primers used in the experiment can be found in Supplementary Table S3. Samples were assayed in duplicate and normalized to endogenous *Gapdh*.

### Neurosphere Assay

Neural progenitors isolated from embryonic brains were seeded in 24-well plate at 2 × 10^5^ cells/ml in SFM with growth factors. After 5 days, the neurospheres were imaged using an inverted microscope. The neurosphere diameter was measured using ImageJ software.

### CFSE Assay

CFSE assay was carried out using the CellTrace CFSE Cell Proliferation Kit according to the manufacturer’s protocol (Thermo). Briefly, neural progenitors isolated from embryonic brains were stain with 5 μM CFSE for 20 min, washed with SFM once, and cultured for 48 h. Subsequently, cells were digested with trypsin and fixed with 2% paraformaldehyde and subjected to flow cytometry analysis.

### BrdU Incorporation Assay

BrdU was added to the primary neural progenitor cell culture at 10 μg/ml. cells were cultured for another 2 h before collected for staining and flow cytometry analysis.

### Intracellular Indirect Immunostaining for Flow Cytometry Analysis

Cells were collected by trypsinization and fixed with 2% paraformaldehyde at 4°C overnight and washed once with intracellular staining buffer (ICSB, 1% FBS in PBS with 0.01% sodium azide). Cells were then permeabilized by 90% ice-cold methanol and washed once with ICSB. (For staining of BrdU, cells were then treated with DNase for 1 h at 37°C to expose the antigen, and washed once with ICSB.) Subsequently, cells were incubated with primary antibody diluted in ICSB for 1hr at room temperature, washed once, followed by fluorophore-conjugated secondary antibody incubation for 30 min. cells were then stained with DAPI or TO-PRO, washed once and subjected to flow cytometry analysis.

### Chromatin Immunoprecipitation Assay

Wild-type neurospheres were crosslinked with formaldehyde at a final concentration of 1% for 10 min followed by quenching with Glycine. Chromatin extracts were fragmented by sonication and precleared with protein G Dynabeads, and subsequently precipitated with anti-STAT3 antibody (Santa Cruz, C20) or normal rabbit IgG (Santa Cruz) overnight at 4°C. After washing and elution, crosslink reversal was done by incubating at 65°C for 8 h. The eluted DNA was purified and analyzed by qPCR with primers specific to the predicted STAT3 binding site. qPCR experiment was carried out using SYBR green qPCR kit from KAPA and Applied Biosystems 7500 Real PCR System. Primer sequences for Chip-qPCR could be found in Supplementary Table S4. Samples were assayed in duplicate.

### Dual-Luciferase Reporter Assay

Promoters of genes of interest were cloned into pGL4.17 vector (Promega) and transfected into Neuro2A cells along with the pRL-TK vector (Promega). Sixteen hours after transfection, cells were treated with DMSO or STAT3 inhibitor STATTIC at 2 μg/ml. After 48 h, cells were lysed with Passive lysis buffer (Promega) and subjected to luciferase assay in a 96-well format in plate readers according to the manufacturer’s protocol. Primer sequences for the generation of reporter vector could be found in Supplementary Table S5.

### Complex I Activity Assay

Complex I activity was measured using the Complex I activity kit from Cayman Chemistry, according to the manufacturer’s protocol. In brief, neurospheres cultured for 4 days were homogenized in Mitochondrial Homogenization Buffer (Cayman) and were mixed with Complex I Activity Assay Buffer, NaN_3_, FF-BSA reagent and loaded into the test 96-well plate. A mix containing NADH and Ubiquinone was added into each well. The plate was immediately placed in a plate reader to measure absorbance at the wavelength of 340 nm every 30 s for 15 min at 25°C. The decrease rate of absorbance, which reflects the decrease in NADH concentration, was determined to be the activity of Complex I. After the experiment, cells from different groups were lysed to analysis the protein level.

### Mitochondrial Membrane Potential and ROS Measurement

Neurospheres cultured for 4 days were dissociated into single cells with Accutase (Thermo Fisher Scientific) and was resuspended in SFM containing 5 μM Mitotracker CMXRed (Thermo Fisher Scientific), or 5 μM Mitosox Red (Thermo Fisher Scientific) or 5 μM CM-DCFDA (Thermo Fisher Scientific), respectively, and incubated in 37°C CO_2_ incubator for 30 min. Cells were then stained with 1 μg/ml Topro-3 as a live-dead indicator, followed by flow cytometry analysis. For microscopic imaging, neural progenitors cultured on poly-D-lysine and laminin coated coverslips were stained with Mitosox Red or DCFDA for 30 min, washed once with PBS, fixed with 4% PFA and mounted with Prolong Gold DAPI mounting media before subjecting to imaging.

### Seahorse OCR/ECAR Assay

Neurospheres cultured for 4 days were dissociated into single cells and seeded into the Seahorse 24-well test plate coated with the cell-tak reagent at the density of 1 × 10^5^ cells/well. Cells were then centrifuged at 200 *g* for 1min to achieve cell attachment. After equilibrated at 37°C incubator (no CO_2_) for 1 h, oxygen consumption rate (OCR) and extracellular acidification rate (ECAR) of cells were analyzed using Seahorse XFe24 machine. The assay medium was SFM supplemented with sodium pyruvate. For Mito-stress assay, OCR and ECAR of cells were measured three times at basal state and after addition of 1 μM oligomycin, 2 μM of FCCP, and a mix of 1 μM Rotenone and 1uM Antimycin A, respectively. After the experiment, cells from different groups were lysed and subjected to western blot experiment to confirm the same seeding density between groups. For glycolysis assay, the ECAR of cells were measured 3 times at basal level and after the addition of 20 mM of Glucose, 1 μM oligomycin, and 50 mM 2DG, respectively.

### ADP/ATP Ratio Assay

ADP/ATP ratio assay was conducted using the ADP/ATP ratio assay kit from Sigma Aldrich (MAK135) according to the manufacturer’s protocol. Briefly, ∼10^4^ cells were seeded in each well of the 96-well plate. After culture for 1 day, the medium was removed and the ATP reagent containing substrate, co-substrate, and ATP enzymes was added to the plate, and luciferase activity was measured (RLU_A_) after 1 min incubation. The luciferase activity was measured again (RLU_B_) after another 10 min incubation to get the basal luminesce before adding the ADP enzymes. Therefore, the ADP enzymes were added to the plate, and the luciferase activity was measured (RLU_C_) after 1min incubation. The ADP/ATP ratio were determined as (RLU_C_-RLU_B_)/RLU_A_.

### Mitochondrial DNA Copy Number Measurement

Neurospheres cultured for 4 days were lysed with RIPA buffer. The lysate was then subjected to qPCR experiment using primer pairs targeted to mitochondrial DNA or genomic DNA. The experiment was carried out with SYBR Green qPCR kit from KAPA and Applied Biosystems 7500 Real PCR System. The mitochondrial DNA copy number was normalized to the genomic DNA copy number. Samples were assayed in duplicate.

### Western Blot

Cells were lysed with RIPA buffer (50 mM Tris-HCl pH7.4, 150 mM NaCl, 1% NP-40, 0.25% Na-deoxycholate and 1 mM EDTA), centrifuged at 20000 g for 10 min and supernatant were collected. Protein concentrations were measured and 20 μg of total protein were loaded into each well in SDS-PAGE. Samples were then transferred to PVDF membrane(Thermo) and immunoblotted with anti-NDUFS3 (Invitrogen), anti-NDUFA13(Invitrogen), anti-SDHA(CST), anti-beta-actin (Santa Cruz), anti-GAPDH (Sigma), anti-STAT3 (CST), anti-pAMPK (CST), AMPK (CST), anti-pS-AKT(CST), anti-AKT(CST), anti-CASP3(CST) anti-p-p38(CST), anti-p38(CST), anti-mTOR(CST), anti-pS-STAT3(CST), anti-pY-STAT3(CST), anti-LC3(CST), anti-GFAP(SCBT), anti-ALDH1L1(Abcam) followed by HRP-conjugated secondary antibody (Thermo) incubation and the SuperSignal West Femto Maximum Sensitivity Substrate (Thermo) was used to detect signal.

### Immunofluorescence

Tissues were isolated and fixed in 4% paraformaldehyde (PFA) in PBS overnight at 4°C. Fixed tissues were dehydrated by incubation in a series ethanol gradient and Xylene, then embedded in paraffin wax for sectioning at the thickness of 5 μm. Slices obtained were rehydrated and boiled in Sodium Citrate solution at 120°C for 20 min for antigen retrieval. Slices were washed in PBS once before the blocking step. Cells cultured on the coverslip were fixed in 4% paraformaldehyde (PFA) in PBS overnight at 4°C. Subsequently, coverslips were washed in PBS three times before blocking.

The brain slices or coverslips were incubated for 1 h in blocking solution containing 5% bovine serum albumin (BSA, Sigma) and 0.1% Triton X-100 (Sigma) in PBS. They were then incubated with primary antibody overnight at 4°C, followed by washing three times in PBS before incubation with fluorophore-conjugated secondary antibodies. After secondary antibody incubation, they were stained with DAPI, washed three times and mounted with Prolong Gold Mounting Medium (Thermo) for imaging. Images were captured using a Zeiss Axio Imager M2 fluorescence microscope. Image analysis was done using ImageJ. The mitochondria morphology was analyzed by ImageJ plugin MiNA (Valente et al., 2017).

### Transfection

Overexpression or knockdown of STAT3 and the OXPHOS genes in cell lines were done by transfection using Lipofectamine 3000 (Thermo Fisher Scientific) according to the manufacturer’s protocol. Briefly, 2.0 μg of plasmid DNA were used for each well of a 6-well plate. DNA was mixed with 7.5 μl of P3000 reagent and 5 μl Lipofectamine3000 in OptiMEM (Thermo Fisher Scientific), incubated for 5 min and added to the cells. The backbone of the overexpression vectors was pMX vector from Addgene, while the shRNA vectors were constructed from pLVX-shRNA2 (Clonetech). The shRNA sequences were obtained from Sigma Aldrich database. The primers for shRNA vectors generation were listed in Supplementary Table S6.

### Lentivirus Preparation and Neural Progenitor Infection

The lentivirus was produced using the 3rd generation system. The virus envelope plasmid and the packaging plasmids, as well as the pLVX-shRNA2 plasmids, were transfected into 293T cell lines. Six hours after transfection, media were changed to the fresh ones. The lentivirus containing media were collected after 24 h and then subjected to ultracentrifugation at 100,000 *g* for 1 h to concentrate the lentivirus particle. The lentivirus particles were then resuspended in SFM and stored at 4°C before used. To infect the neural progenitors, the primary neural progenitors were dissociated from neurospheres via multiple pipetting. Cells were then seeded in the lentivirus-containing SFM at 2 × 10^5^ cells/ml.

### Drug Treatment

For the STA-21 treatment of neural progenitors, STA-21 was added to cell culture at 2 μg/ml. After 48 h, neural progenitors were collected for RNA extraction or subjected to cell differentiation.

For the investigation of the effect of ROS on proliferation, neural progenitors were treated with 10 μM H_2_O_2_, or 20 mM NAC or DMSO control. To investigate the effect of AMPK and autophagy pathway, neural progenitors were treated with 4 mM 2-deoxyglucose (2DG), 1 μM Metformin, 2 mM 3 mA, or 2.5 μM Compound C.

### Statistics

Statistical significance was determined by Student’s *t*-test or Mann–Whitney test using GraphPad Prism 6.01. The *p*-value < 0.05 was considered significant. Unless otherwise specified, data were presented as mean and the standard deviation (mean ± SD).

## Results

### Stat3 Deletion in Neural Progenitors Resulted in Faster Proliferation and Reduced Neurogenesis

**FIGURE 1:**
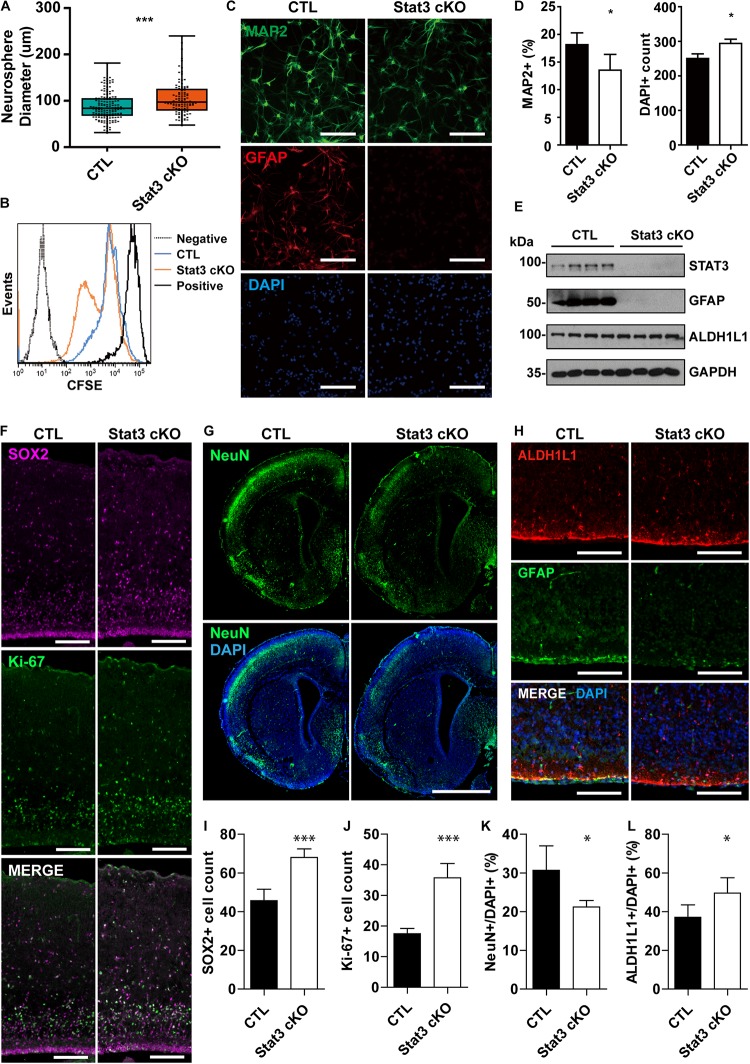
*Stat3* cKO in neural progenitors leads to increased proliferation rate and decreased neuronal differentiation. **(A)** Neurosphere assay showed that Stat3 cKO neural progenitor has a higher proliferation rate. Box plot represents the median, maximum and minimum value as well as the interquartile range. Mann–Whitney test was used to determine the statistical significance. **(B)** CFSE showed that Stat3 cKO cells divided faster than control cells (C; F/ +). **(C,D)** Immunofluorescence staining of MAP2 and GFAP in differentiated control and *Stat3* cKO neural progenitors. Statistical significance was determined using the student t-test. Error bars represent mean ± standard deviations. **(E)** Western blot of differentiated neural progenitor samples showed that *Stat3* cKO led to the loss of GFAP expression, but the ALDH1L1 expression was not altered. **(F)** Immunostaining of radial glia marker SOX2 and proliferation marker Ki67 in E17 mouse brain. Scale Bar: 100 μm. **(G)** Immunostaining of NeuN in P1 mouse brain. Scale bar: 250 μm. **(H)** Immunostaining of GFAP and ALDH1L1, both astrocyte markers, in the P1 mouse brain. Scale bar: 20 μm. **(I–L)** Cell counts and percentages obtained from **(F–H)**. The cell numbers were quantified from five images, respectively. Error bars represent mean ± standard deviations. ^∗∗∗^*p* < 0.001, ^∗^*p* < 0.05.

In this study, we investigated the function of STAT3 in neural progenitors using the Nestin-cre; Stat3-flox mice, which achieves *Stat3* conditional KO in neural progenitors by E14 (Tronche et al., 1999; Moh et al., 2007) (Supplementary Figure S1). To determine the effect of STAT3 on neural progenitor proliferation, we carried out the primary neurosphere growth assay on neural progenitors isolated from E15.5 embryos. Intriguingly, we found that deletion of *Stat3* in neural progenitor cells leads to an increase of neurosphere size compared to the control (CTL), indicating a faster cell growth (Figure 1A). In addition, the CFSE proliferation assay further confirmed an accelerated mitosis pace in *Stat3* cKO neural progenitors (Figure 1B) compare to control neural progenitors, suggesting a potential role of STAT3 as a negative regulator of neural progenitor proliferation. Consistently, we found that the developing cortex of *Stat3* cKO contains higher numbers of Sox2+ and Ki-67+ cells compared to the CTL, indicating that *Stat3* deletion promotes self-renewal and proliferation *in vivo* (Figures 1F,I,J).

To examine how *Stat3* deletion affects the differentiation potential of neural progenitors, we quantified the MAP2^+^ neurons and the GFAP^+^ astrocytes in the differentiated CTL and *Stat3* cKO neural progenitors. We found that conditional deletion of Stat3 resulted in fewer MAP2^+^ neuron generation and abolished astrocyte differentiation associated GFAP expression (Figures 1C,D). Immunostaining experiment of postnatal day 1 brain slices also demonstrated that *Stat3* cKO leads to decreased neuronal differentiation, as was shown by the decreased NeuN+ cell percentage (Figures 1G,K).

To examine if astrogenesis is indeed inhibited in *Stat3* cKO, we analyzed the expression of the pan-astrocyte marker ALDH1L1, along with GFAP, in differentiated neural progenitors. Interestingly, ALDH1L1 expression was not affected by *Stat3* cKO, while that of GFAP was abolished (Figure 1E). Similarly, we found that *Stat3* cKO generated a higher percentage of ALDH1L1+ cells compared to the CTL (Figures 1H,L). This suggested that STAT3 is not necessary for astrogenesis but negatively regulates it instead.

### Expression Profile Analysis of Stat3 cKO in Neural Progenitor Cells

**FIGURE 2:**
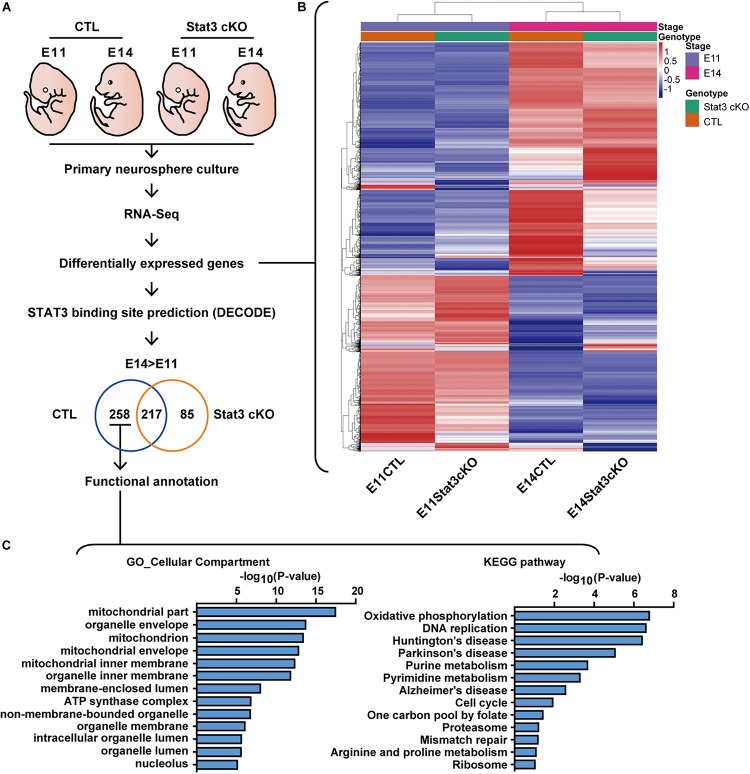
RNA-seq revealed that STAT3 promotes the expression of genes involved in mitochondrial metabolism. **(A)** Experiment scheme of RNA-seq experiment. **(B)** Heatmap of the differentially expressed gene between four experiment groups. **(C)** Functional annotation of genes upregulated between E11 and E14 in CTL compared to *Stat3* cKO.

To gain insight into the downstream pathway of STAT3 in neural progenitors, we performed RNA-Seq analysis on the CTL and *Stat3* cKO neural progenitors isolated from E11 or E14 embryos. For the expression analysis, the RNA-seq data were processed using the Tophat-Cufflinks workflow (Trapnell et al., 2012), then clustered according to their expression level differential in the CTL and *Stat3* cKO populations at the E11 and E14 stages. To filter for potential direct target genes of STAT3, the genes that were differentially expressed were cross-referenced with the DECODE database to identify those with the potential STAT3 binding sites (Figures 2A,B). Genes that were uniquely upregulated in control cells from E11 to E14 compare to that of *Stat3* cKO cells are potentially STAT3 target genes, which were further analyzed using the gene-set enrichment tool, DAVID, for gene annotation and pathway enrichment analysis (Huang da et al., 2009a, b) (Figure 2C).

Functional annotation of the potential STAT3 target genes by cellular compartment (GO_CC) localized a significant set of enriched genes to the mitochondria. Consistent with this finding, annotation with Kyoto Encyclopedia of Genes and Genomes (KEGG) pathway revealed that the putative STAT3 target genes are mainly enriched in the Oxidative phosphorylation (OXPHOS) pathway, and to a lesser extent in other pathways such as purine metabolism, pyrimidine metabolism, and one carbon pool by folate. These genes were also enriched in pathways associated with neurodegenerative diseases such as Huntington’s disease, Parkinson’s disease and Alzheimer’s disease (Figure 2C).

Given our findings that STAT3 is required for the differentiation of neurons and astrocytes, we next examined the expression of marker genes of different cell types, such as radial glia, intermediate progenitor cells (IPCs), neurons and astrocytes. We found that compared to the CTL, *Stat3* cKO neural progenitors showed increased expression of radial glia markers such as *Sox2*, *Pax6*, and *Sfrp2*, and decreased expression of most IPC and neuronal markers (Supplementary Figures S2A–C). The majority of glial markers such as *Aqp4*, *Aldh1l1*, and *Mfge8* were upregulated in *Stat3* cKO progenitors, while *Gfap* expression was suppressed (Supplementary Figure S2D). These results support our *in vitro* findings that *Stat3* loss-of-function led to decreased neuron differentiation.

Interestingly, *Stat3* cKO also resulted in the increased expression of upper layer (layer II-III) neuron markers such as *Cux1*, *Cux2*, but decreased expression of deeper layer (layer IV-VII) markers such as *Ctip2* and *Tbr1*. This result is consistent with previous reports by Hong et al. that *Stat3* cKO resulted in a larger number of upper layer neuron generation, suggesting a possibility that STAT3 might function to regulate layer specification during cortical development (Hong and Song, 2015) (Supplementary Figure S2E).

### STAT3 Promotes the Expression of Oxidative Phosphorylation (OXPHOS) Genes in Neural Progenitor Cells

**FIGURE 3:**
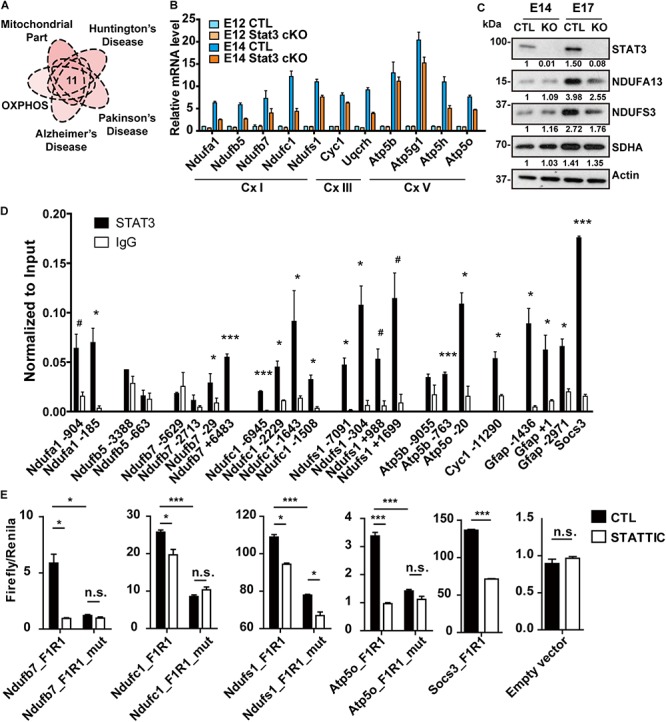
STAT3 promotes the expression of OXPHOS genes in neural progenitors. **(A)** One group of potential STAT3 targets was involved in the mitochondrial oxidative phosphorylation as well as the neurodegenerative diseases. **(B)** RT-qPCR confirmed the expression of these genes was upregulated during neural progenitor development and downregulated in *Stat3* cKO. **(C)** Western blot showed that the expression of complex I proteins was downregulated in *Stat3* cKO neural progenitors isolated at E17. **(D)** ChIP-qPCR showed that STAT3 directed bind to the promoter or enhancer of the OXPHOS genes such as *Ndufa1*, *Ndufb7*, *Ndufc1*, *Ndufs1*, *Atp5b*, *Atp5o*. **(E)** Dual luciferase reporter assay showed that STAT3 binds to the promoter of OXPHOS genes and activate their transcription. Treatment of STATTIC or mutation of the STAT3 binding site downregulated the reporter activity. Socs3 promoter was used as the positive control, while the empty reporter vector was used as the negative control. #*p* < 0.08, *p < 0.05, **p < 0.01, ***p < 0.005, n.s. not significant. Error bars represent mean ± standard deviations.

RNA-seq analysis revealed a group of putative STAT3 targets that not only involves in the OXPHOS pathways, but is also implicated in neurodegenerative diseases (Figures 2C, 3A). Previous studies have demonstrated that OXPHOS genes are highly expressed in the VZ in E14.5 mouse brain (Visel et al., 2004), suggesting the governing of OXPHOS genes expression may be critical during brain development (Supplementary Figure S3A).

To validate our RNA-seq results showing differential expression of OXPHOS genes, we performed quantitative reverse-transcription PCR (RT-qPCR) to compare expression of selected genes, including complex I genes (*Ndufa1*, *Ndufb5*, *Ndufb7*, *Ndufc1* and *Ndufs1*), complex III genes (*Cyc1* and *Uqcrh*), as well as complex V genes (*Atp5b*, *Atp5g1*, *Atp5h*, and *Atp5o*), in CTL and *Stat3* cKO neural progenitors at E12 and E14. In agreement with the RNA-seq analysis, we found that the expression of these putative STAT3 target OXPHOS genes was significantly upregulated in E14 CTL neural progenitors, and significantly downregulated in E14 *Stat3* cKO neural progenitors (Figure 3B). However, the protein level of OXPHOS genes was not altered in Stat3 cKO neural progenitors at E14. We speculated that the change in the protein level might become evident in the later stage due to delayed response of protein turnover. Indeed, the OXPHOS protein level was downregulated in Stat3 cKO neural progenitors at E17 (Figure 3C).

To further confirm that STAT3 regulate the OXPHOS gene expression, we examined the expression of OXPHOS genes in neural cells with drug/genetic-modulated STAT3 activity. Expression of these OXPHOS genes was downregulated under treatment of STA-21 in E14 neural progenitors (Supplementary Figure S3B). In addition, the deletion of *Stat3* with CRISPR in ESC-derived mouse neural stem cells led to the downregulation of OXPHOS genes, while overexpression of STAT3 in Neuro2A cells upregulated OXPHOS gene expression (Supplementary Figures S3C,D). Taken together, these results strongly support that STAT3 is indispensable for the transcription regulation of OXPHOS genes during neurogenesis.

Next, we investigated whether STAT3 directly regulates the expression of the OXPHOS genes. By performing ChIP-qPCR assays using E14 WT neural progenitors, we found that STAT3 bound to the promoter or enhancer loci of a significant proportion of the target OXPHOS genes (Figure 3D). In addition, unphosphorylated STAT3 has a higher affinity to some of these loci and promotes gene expression, compared to the tyrosine-phosphorylated STAT3 (Supplementary Figure S4). Luciferase reporter assays with selected promoter/enhancer regions in Neuro2A cells further demonstrated that treatment of STAT3 inhibitor Static or mutation of the putative STAT3 binding sites in those regions significantly attenuates the reporter activity (Figure 3E). Taken together, these results support the hypothesis that STAT3 promotes the expression of OXPHOS gene via direct transcriptional regulation.

### STAT3 Promotes Mitochondria Respiration in Neural Progenitors

**FIGURE 4:**
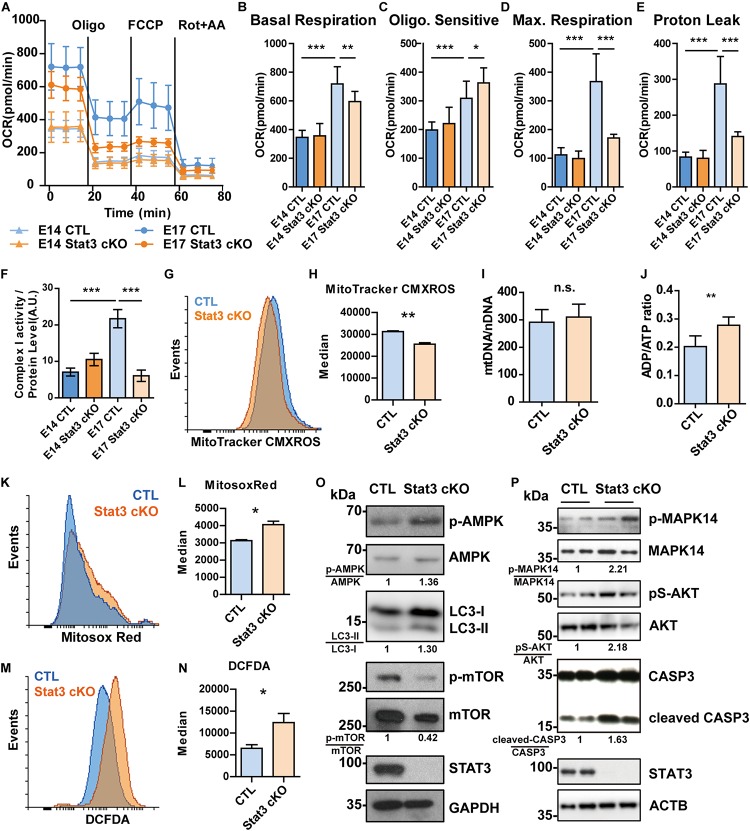
STAT3 regulates mitochondrial respiration and ROS production in neural progenitors. **(A–E)** OCR of E14 neural progenitors was comparable between CTL and *Stat3* cKO. But at E17, the OCR of *Stat3* cKO was significantly decreased compared to the CTL. **(F)** The Complex I activity was compromised in E17 *Stat3* cKO neural progenitors. **(G,H)** E17 *Stat3* cKO neural progenitors had lower mitochondrial membrane potential indicated by MitoTracker CMXROS staining. **(I)** qPCR showed that the ratio of mitochondrial-DNA to nuclear-DNA is not significantly changed between CTL and *Stat3* cKO neural progenitors. **(J)** ADP/ATP ratio assay showed that *Stat3* cKO neural progenitors had elevated ADP/ATP ratio. **(K,L)** Flow cytometry showed that *Stat3* cKO neural progenitors had increased mitochondrial superoxide production. **(M,N)** Flow cytometry showed that *Stat3* cKO neural progenitors contained increased cellular ROS. **(O)** Western blot showed that *Stat3* cKO neural progenitors had elevated AMPK pathway activation accompanied by decreased mTOR. **(P)** *Stat3* cKO resulted in increased activation of MAPK14 and AKT, as well as increased CASP3 cleavage. Each lane was sample collected from individual embryo of the same little. Oligo, oligomycin; Rot, Rotenone; AA, antimycin A. Error bars represent mean ± standard deviations. *p < 0.05, **p < 0.01, ***p < 0.005, n.s. not significant.

Given that OXPHOS genes are involved in mitochondrial respiration, we next examined whether *Stat3* loss-of-function would alter the oxygen consumption rate (OCR). We found that, at E14, the basal OCR of *Stat3* cKO and CTL neural progenitors remains indistinguishable. However, at E17, the increment of basal OCR, Maximum OCR and Proton Leak in *Stat3* cKO neural progenitors is significantly hindered when compare with that of the CTL cells (Figures 4A–E). These results suggest that there is a demand for energy metabolic increases in neural progenitors from E14 to E17, and STAT3 is a critical drive during this process.

As several of the candidate STAT3 target OXPHOS genes are components of complex I, we examined the complex I activity and found that *Stat3* loss-of-function decreased the activity of complex I at E17 (Figure 4F). In addition, we found that this impaired complex I activity was associated with a decrease in mitochondrial membrane potential (MMP) (Figures 4G,H). To exclude the possibility that the decrease of mitochondrial complex I activity was due to mitochondrial functional alternation, we examined the mitochondrial copy number by qPCR, and found that there were no significant differences between the CTL and *Stat3* cKO cells (Figure 4I).

As mitochondrial respiration is crucial for cellular energy balance, we next examined the ADP/ATP ratio and the related signaling pathways in *Stat3* cKO and control neural progenitors. We found that *Stat3* cKO cells had an elevated ADP/ATP ratio (Figure 4J), which correlated with increased activation of AMPK, the energy balance sensor (Figure 4O). Concomitantly, we found that the phosphorylation of mTOR was decreased in *Stat3* cKO cells. This suggested that the change in mitochondria energy production altered signaling pathways linked to energy and nutrient sensing.

Finally, we found that *Stat3* cKO neural progenitors showed decreased glycolytic flux, which is in line with the observation of the decrease expression of the majority of glycolytic genes (Supplementary Figures S5A–D). Accordingly, we noted a decrease in the expression of pentose phosphate pathway genes that are closely linked with glycolysis (Supplementary Figure S5F). Conversely, the expression of genes involved in fatty acid beta-oxidation was increased suggesting a possible remodeling of metabolic fluxes in *Stat3* cKO (Supplementary Figure S5E).

### STAT3 Negatively Regulates Reactive Oxygen Species (ROS) Production in Neural Progenitors

Whereas abnormal OXPHOS complex activity and MMP can affect mitochondrial ROS production (Murphy, 2009), we next sought to investigate whether the deletion of *Stat3* affects ROS production. We found that the deletion of STAT3 promoted the production of mitochondrial superoxide and cellular ROS level (Figures 4K–N and Supplementary Figures S6D,E). Interestingly, Mitosox-Red-stained mitochondria were elongated in *Stat3* cKO cells, compared to the CTL cells, suggesting a fusion-fission defect in *Stat3* cKO neural progenitors (Supplementary Figures S6A–C).

As ROS signaling can activate different signaling pathways including PI3K/AKT (Lee et al., 2002; Leslie et al., 2003; Lim and Clement, 2007), we next sought to determine the activation status of key signaling pathways linked to ROS. We found that the AKT pathway was activated in *Stat3* cKO neural progenitors, as well as the p38 MAPK (MAPK14) pathway (Figure 4P). Similarly, *Stat3* cKO led to a higher level of cleaved Caspase3 (Figure 4P). However, Stat3 cKO did not result in increased apoptosis in neural progenitors (Supplementary Figure S7).

### Knockdown of Putative STAT3-Target OXPHOS Genes Mimics the Phenotypes of *Stat3* cKO

**FIGURE 5:**
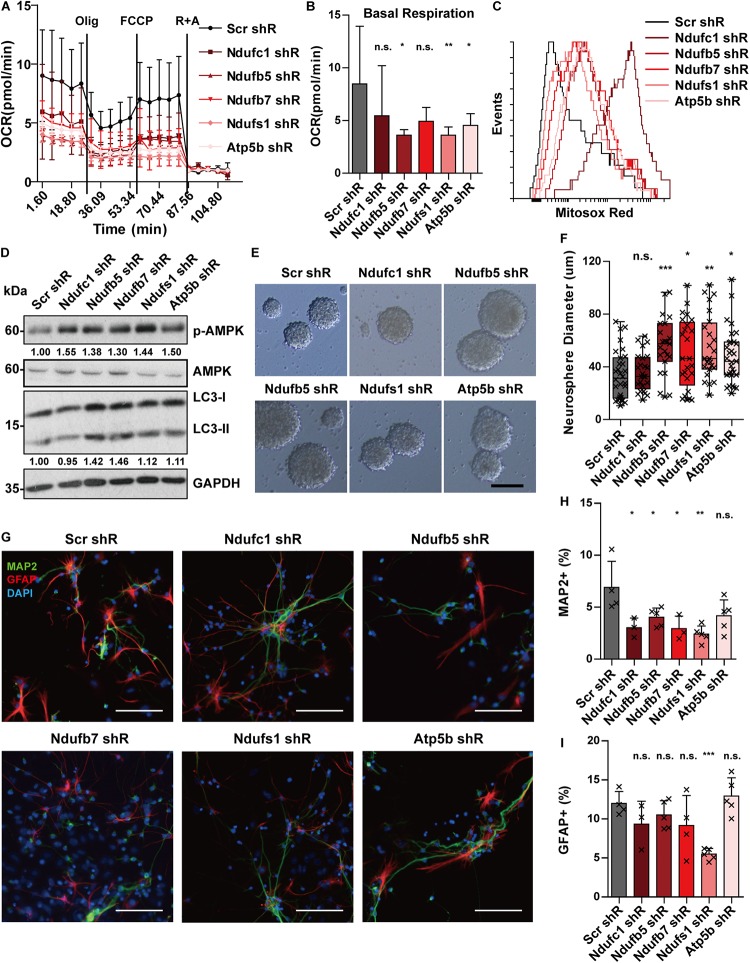
Knockdown of OXPHOS genes mimics the phenotypes of Stat3 cKO. **(A,B)** Knockdown of OXPHOS genes downregulated OCR of neural progenitors. Error bars represent mean ± standard deviations. *p < 0.05, **p < 0.01, ***p < 0.005, n.s. not significant. **(C)** Knockdown of OXPHOS genes promoted mitochondria superoxide production. **(D)** Knockdown of OXPHOS genes activated the AMPK pathway and the autophagy marker. **(E–H)** Knockdown of OXPHOS genes (except Ndufc1) led to a significantly higher proliferation rate of neural progenitors. Scale bar: 50 μm. Box plot represents the median, maximum and minimum value as well as the interquartile range. Mann–Whitney *U* test was used to determine the statistical significance of the neurosphere size difference. *p < 0.05, **p < 0.01, ***p < 0.005, n.s. not significant. **(G–I)** Knockdown of OXPHOS genes resulted in decreased neurons and astrocytes generation. Error bars represent mean ± S.E.M. Knockdown efficiency was validated by RT-qPCR (Supplementary Figure S8).

We previously showed that *Stat3* loss-of-function can downregulate target OXPHOS genes and this correlated with a decrease in mitochondrial function. To determine whether the defects in mitochondrial respiration in STAT3 cKO neural progenitors are mediated primarily by the downregulation of OXPHOS gene expression, we attempted to knockdown several of the target genes (including *Ndufc1*, *Ndufb5*, *Ndufb7*, *Ndufs1*, and *Atp5b*) in E15 neural progenitors using a lentiviral shRNA system. We found that knockdown of the selected target genes led to decreased mitochondrial OCR and increased mitochondrial ROS production, suggesting that downregulation of the target OXPHOS genes primarily mediates the mitochondrial metabolic change observed in Stat3 loss-of-function (Figures 5A–C). Consistently, knockdown of the OXPHOS genes led to the activation of the AMPK pathway and increased autophagy (Figure 5D).

The results above demonstrate that STAT3 is necessary to promote nuclear-encoded OXPHOS gene expression and thereby mitochondria respiration in neural progenitors. To examine another possibility of whether STAT3 regulates the OXPHOS genes expression via mitochondria metabolism, we used 2DG or H_2_O_2_ intervention to mimic the metabolic stress in *Stat3* cKO cells. We found that the OXPHOS genes expression was increased as a result of the drug treatment, in contrast to that in *Stat3* cKO (Supplementary Figure S9). This suggested that in *Stat3* cKO neural progenitors, downregulation of the OXPHOS genes expression leads to the disruption of mitochondrial metabolism, not *vice versa*.

We next sought to determine whether the downregulation of OXPHOS genes mediates the proliferation and differentiation phenotype of *Stat3* cKO neural progenitors. We found that knockdown of these OXPHOS genes led to increased cell proliferation, suggesting that the proliferative phenotype of *Stat3* cKO neural progenitors could be directly mediated by decreased expression of OXPHOS genes (Figures 5E,F). In addition, we found that knockdown of the OXPHOS genes in neural progenitor cells led to significantly lower neuronal differentiation, except that of *Atp5b* (Figures 5G,H). However, knockdown of the selected OXPHOS genes other than *Ndufs1* did not alter the astrogenesis of neural progenitors (Figures 5G,I).

In summary, knockdown of OXPHOS genes was able to reproduce several phenotypes of *Stat3* cKO neural progenitor cells, including reduced OCR, increased ROS production, and activation of the AMPK pathway. In addition, the knockdown of OXPHOS genes resulted in an increased proliferation rate and reduced neurogenesis, similar to the *Stat3* cKO neural progenitors. These findings support the hypothesis that the regulation of OXPHOS genes mediates the phenotypic changes observed in *Stat3* loss-of-function neural progenitors.

### Activation of AMPK and ROS Pathways Promotes Neural Progenitor Proliferation

As mitochondria dysfunction in *Stat3* cKO activated the AMPK and ROS pathway, we wondered if these pathways mediated the hyperproliferative phenotype of *Stat3* cKO neural progenitors.

**FIGURE 6:**
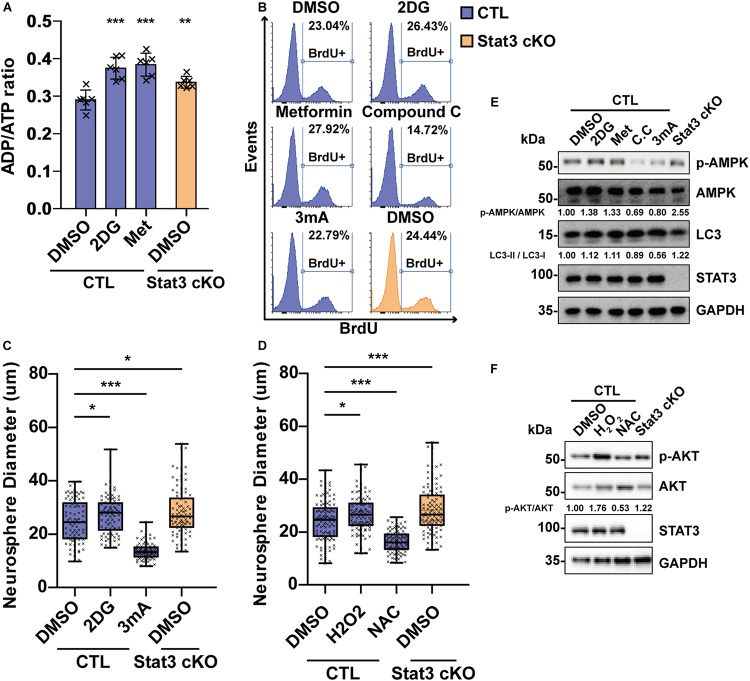
Modulation of energy production and ROS level regulates neural progenitor proliferation. **(A)** ADP/ATP ratio change in CTL and *Stat3* cKO neural progenitors under 2DG and Metformin treatment. Met, Metformin. Error bars represent mean ± standard deviations. **(B)** BrdU incorporation assay on CTL and *Stat3* cKO neural progenitors in various treatments. **(C)** Neurosphere assay showed that 2DG treatment promoted the proliferation of neural progenitors, while 3 mA treatment inhibited cell proliferation. **(D)** Neurosphere assay on CTL and *Stat3* cKO neural progenitors treated with H_2_O_2_ or NAC. Error bars represent mean ± standard deviations. Box plot represents the median, maximum and minimum value as well as the interquartile range. *p < 0.05, **p < 0.01, ***p < 0.005, n.s., not significant. **(E)** Western blot of neural progenitors treated by 2DG, Metformin (Met), Compound C (C.C) and 3 mA. **(F)** Western blot of neural progenitors treated by H_2_O_2_ and NAC.

To investigate the role of AMPK in neural progenitor proliferation, we treated E15 neural progenitors with AMPK agonists 2DG and Metformin as well as AMPK suppressors Compound C and inhibitor of downstream autophagy 3 mA. Treatment of 2-deoxyglucose (2DG) and Metformin in CTL neural progenitors induced a starvation state similar to that of *Stat3* cKO, as was dictated by the ADP/ATP ratio (Figure 6A). 2DG and Metformin treatment resulted in increased neurosphere size and BrdU incorporation in CTL neural progenitor, while treatment of Compound C and 3mA decreased the neurosphere size and BrdU incorporation (Figures 6B,C), suggesting that AMPK pathway activation is both sufficient and necessary for neural progenitor proliferation. To investigate whether ROS signaling regulates neural progenitor proliferation, H_2_O_2_ and ROS scavenger *N*-acetylcysteine (NAC) was added to the CTL neural progenitors. Treatment of H2O2 led to increased BrdU incorporation and neurosphere growth, while NAC treatment decreased them, suggesting that ROS signaling is also sufficient and necessary for neural progenitor proliferation (Figure 6D). Modulation of AMPK pathway and ROS downstream pathway AKT by drug treatment was verified by western blot analysis (Figures 6E,F).

## Discussion

STAT3 has been shown to play an important role in neural progenitor development, but its function and downstream pathways are not fully understood. Our study discovers a new role of STAT3 and sheds light on the downstream regulatory network. We found that STAT3 is necessary for controlling the proliferation rate and promoting neurogenesis via a mitochondria-dependent pathway.

Previous studies found activation of STAT3 by cytokines promotes neural progenitor proliferation and negatively regulates neurogenesis (Hong and Song, 2015). However, the effect of cytokines could be independent of the JAK-STAT3 pathway. For instance, CNTF treatment induced neural progenitor proliferation and suppressed neurogenesis in both CTL and *Stat3* cKO neural progenitors (data not shown). Our study showed that loss of STAT3 also results in increased proliferation and reduced neurogenesis. This is consistent with previous report that *Stat3* cKO astrocytes resulted in increased proliferation (de la Iglesia et al., 2008).

Additionally, we found that STAT3 is not necessary for astrocytogenesis. Previous studies showed that STAT3 is necessary for astrocyte marker GFAP expression (Hong and Song, 2014; Kanski et al., 2014). Several STAT3 binding sites were found in *Gfap* promoter (Bonni et al., 1997; Nakashima et al., 1999), and their interaction with STAT3 is critical for *Gfap* expression. However, GFAP is merely a marker of a subset of astrocytes (Zeisel et al., 2015). Lack of GFAP+ cells in *Stat3* cKO suggests that it leads to potentially functional tampered astrocytes, but not the reduction of the astrocyte cell number. On the contrary, expression of other astrocyte markers was even increased in *Stat3* cKO, which might due to increased proliferation of astrocyte precursors.

Our RNA-seq data showed that expression of upper layer neuronal marker was increased in *Stat3* cKO neural progenitors, which was concomitant with the observation in previous study that *Stat3* cKO yielded significantly increased number of upper layer (layer II–III) neurons (Hong and Song, 2015). Upper layer neurons are generated after E14, when *Stat3* cKO neural progenitors start to show defect of mitochondrial respiration and increased proliferation rate (Gao et al., 2014). Therefore, increased upper layer neuron generation might result from the increased proliferation of neural progenitors. In addition, whether mitochondria respiration correlates with different layer neuron specification remains to be studied. Electron microscopic study suggested that layer II–III neurons contain less volume of mitochondria compared to layer IV (Santuy et al., 2018), implying that neurons in different cortical layers might have different mitochondrial metabolism requirement.

In the canonical JAK-STAT pathway, STAT3 is tyrosine phosphorylated, undergoes nucleus import and activates gene expression (Fu et al., 1990, 1992; Schindler et al., 1992; Darnell et al., 1994; Levy and Darnell, 2002). However, STAT3 without tyrosine-phosphorylation (U-STAT3) could also promote gene expression. It has been reported that U-STAT3 could be imported into nucleus by Importin (Liu et al., 2005; Cimica et al., 2011). In addition, U-STAT3 could accumulate in the nucleus after prolonged IL6 treatment (Yang et al., 2005). U-STAT3 could interact with DNA and promote gene expression by forming homodimer, or in a complex with RELA (Yang et al., 2007; Timofeeva et al., 2012; Nkansah et al., 2013; Okada et al., 2018). Similarly, our data showed that overexpression of STAT3-CYF, a dominant negative mutant of STAT3 that mimic U-STAT3, could bind to the regulatory element of Ndufc1 and promote its expression, while the overexpression of STAT3-CA, a constitutive active form of STAT3, could not. However, it remains unclear if other post-translational modifications of STAT3 regulate these genes expression. Furthermore, it was speculated that U-STAT3 might also function as a chromatin scaffold protein due to the long distance of their adjacent binding sites. Indeed, bisulfite sequencing revealed that the global DNA methylation level was decreased in *Stat3* cKO neural progenitors at E14. STAT3 activation could promote cell proliferation and suppress neurogenesis, while absence of STAT3 also leads to similar outcome, suggesting that the STAT3 activation or inactivation might be one of the molecular switch of neural progenitor fate.

It has been shown that STAT3 could be translocated into mitochondria, and that mitochondrial STAT3 promotes respiration by binding to protein complexes such as Complex I, or by regulating mitochondrial transcription (Wegrzyn et al., 2009; Macias et al., 2014; Carbognin et al., 2016; Xu et al., 2016). However, we have previously reported that STAT3 does not locate within mitochondria, but in mitochondria-associated ER membrane instead (Su et al., 2019), which is neglected by prior studies. Even if STAT3 is present in mitochondria, it is unlikely to directly regulate mitochondrial respiration, due to the stoichiometric difference between STAT3 and the OXPHOS complexes or the mitochondrial genome (the molecular ratio of Complex I and STAT3 is ∼10^5^ by estimation; and that of the mitochondrial genome and STAT3 is ∼10^2^) (Bogenhagen and Clayton, 1974; Shmookler Reis and Goldstein, 1983; Phillips et al., 2010). Thus, STAT3 might regulate mitochondrial respiration via other pathways. Our study showed STAT3 can promote mitochondrial respiration and limit ROS production by regulating the expression of nuclear-encoded OXPHOS genes during neural progenitor development. Similarly, it was shown that STAT3 regulates citrate synthase expression so as to promote mitochondrial metabolism in lymphocytes (MacPherson et al., 2017). Taken together, our results suggest a STAT3-regulated nuclear-transcription-dependent pathway that modulates mitochondrial metabolism.

Mitochondrial metabolism is closely associated with neural progenitor self-renewal and differentiation. Our data showed downregulation of OXPHOS genes in *Stat3* cKO leads to a disruption of mitochondrial metabolism, which promotes the proliferation of neural progenitors while negatively regulates neurogenesis, consistent with a previous study (Bartesaghi et al., 2015). In addition, mitochondrial metabolism could regulate neural progenitor proliferation and differentiation via activation of AMPK signaling or ROS-AKT signaling (Dasgupta and Milbrandt, 2009; Le Belle et al., 2011; Williams et al., 2011). Consistently, we found overactivation of both AMPK and AKT pathways in *Stat3* cKO neural progenitors. Though both are necessary for promoting neural progenitor proliferation, the AMPK and AKT signaling has an antagonistic effect on mTOR activation and the downstream response. (Gwinn et al., 2008; Zhao et al., 2017). The downregulated mTOR pathway in *Stat3* cKO neural progenitors we show here suggests the negative regulation by AMPK pathway might play a dominant role which is consistent with the proliferation phenotype, given that activation of mTOR complex 1 pathway negatively regulates neural progenitor self-renewal and promotes differentiation (Magri et al., 2011; Hartman et al., 2013). However, further study is required to understand how these metabolic-related pathways converge onto the neural progenitor cell fate.

In summary, we discovered a STAT3-mitochondria metabolism axis that regulates neural progenitor proliferation and differentiation.

## Data Availability Statement

The raw data generated for this study can be found in the GEO, accession number GSE148564.

## Ethics Statement

The animal study was reviewed and approved by NUS Institutional Animal Care and Use Committee.

## Author Contributions

YS, KB, CY, and YZ conceived and designed the project. YS performed most of the experiments and analyzed the data. WZ established the mouse model. YZ and JZ performed the RNA-seq experiment. CP and TM analyzed the RNA-seq data. ZM and JX provided technical assistance. YS wrote the manuscript. WZ, KB, CY, and YZ revised the manuscript.

## Conflict of Interest

The authors declare that the research was conducted in the absence of any commercial or financial relationships that could be construed as a potential conflict of interest.
