# Supplementary material for: STAT3 Regulates Mouse Neural Progenitor Proliferation and Differentiation by Promoting Mitochondrial Metabolism
Source: Front Cell Dev Biol. 2020 May 19;8:362. doi: 10.3389/fcell.2020.00362 (PMC7248371; doi:10.3389/fcell.2020.00362)
Supplement: Supplementary file 1 [file Presentation_1.pdf]

Supplemental Information

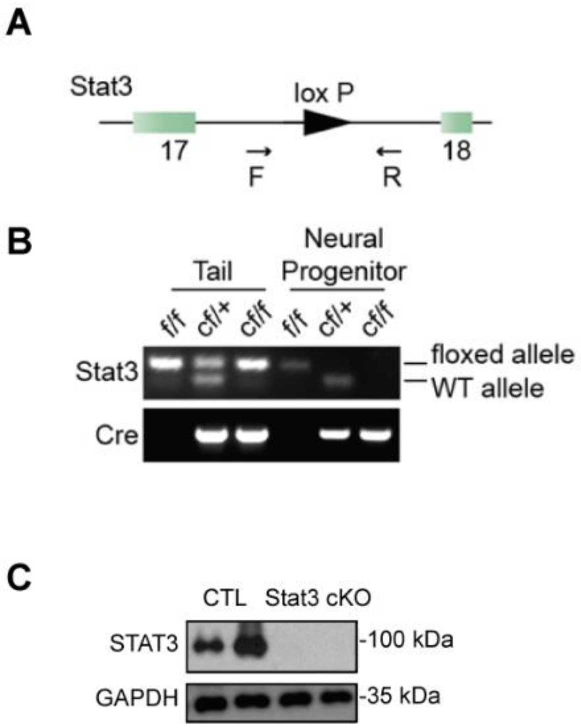

Supplementary Figure S1 Confirmation of successful deletion of *Stat3* exons in neural progenitors. **(A)** Diagram of the genotyping primers. **(B)** Genotyping results in tail or neural progenitor lysates from three different genotypes. **(C)** Western blot showed the expression of STAT3 protein in CTL and *Stat3* cKO neural progenitors.

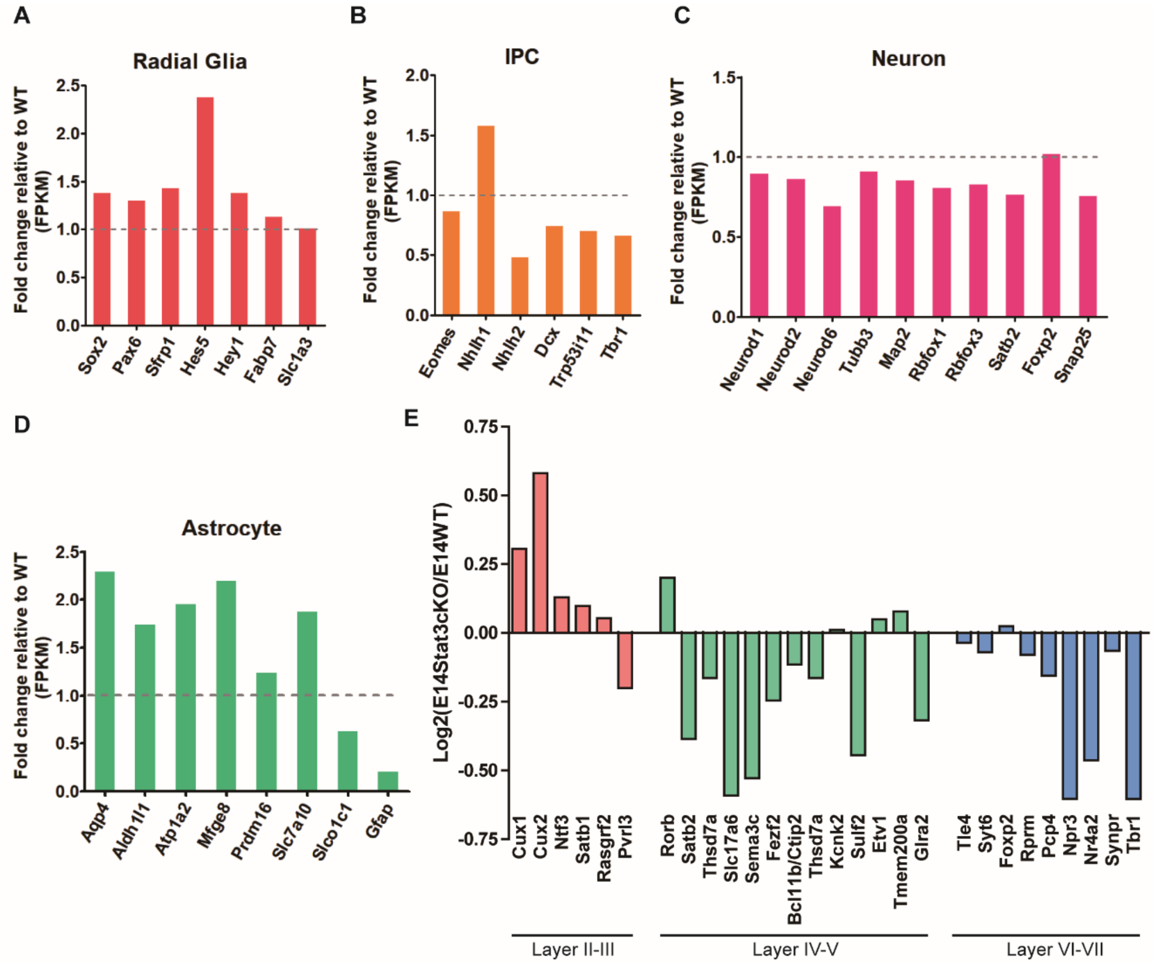

Supplementary Figure S2 Relative expression of neural markers in *Stat3* cKO neural progenitors in E14 revealed by RNA-seq. **(A)** *Stat3* cKO resulted in increased expression of radial glia markers. **(B)** *Stat3* cKO resulted in decreased expression of IPCs markers. **(C)** *Stat3* cKO led to decreased expression of neuron markers. **(D)** *Stat3* cKO led to increased expression of majority of astrocyte markers. **(E)** *Stat3* cKO neural progenitors had increased expression of outer layer neuron markers (II-III) and decreased expression of deeper layer (IV-VII) neuron markers.

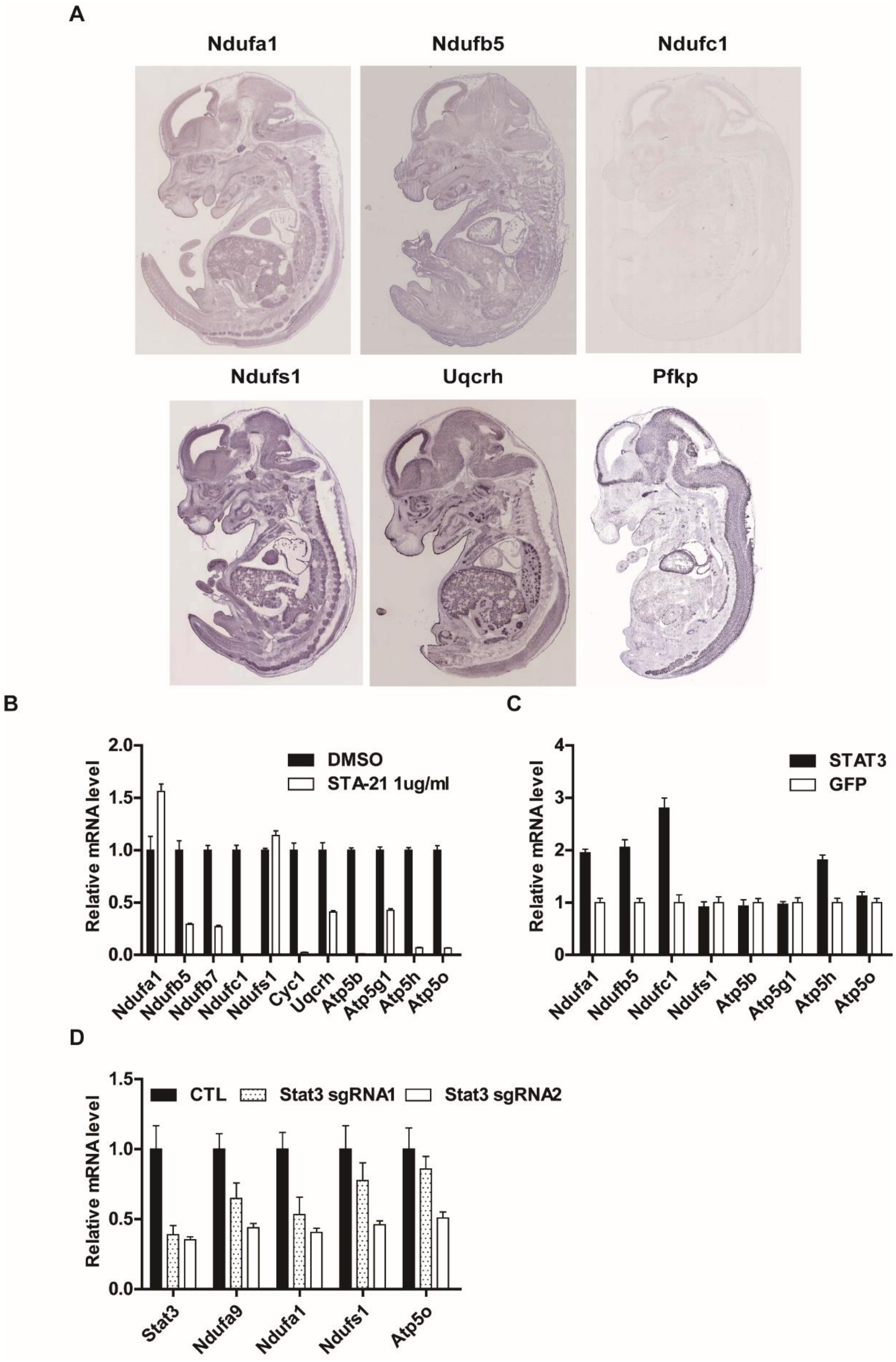

A
Ndufa1
Ndufb5
Ndufc1
Ndufs1
Uqcrh
Pfkp
B
Relative mRNA level
DMSO
STA-21 1ug/ml
Ndufa1
Ndufb5
Ndufb7
Ndufc1
Ndufs1
Cyc1
Uqcrh
Atp5b
Atp5g1
Atp5h
Atp5o
C
Relative mRNA level
STAT3
GFP
Ndufa1
Ndufb5
Ndufc1
Ndufs1
Atp5b
Atp5g1
Atp5h
Atp5o
D
Relative mRNA level
CTL
Stat3 sgRNA1
Stat3 sgRNA2
Stat3
Ndufa9
Ndufa1
Ndufs1
Atp5o

Supplementary Figure S3 STAT3 is both sufficient and necessary to promote expression of OXPHOS genes. **(A)** Expression of OXPHOS genes is enriched in the VZ of E14.5 embryonic brain. In contrast, expression of the glycolysis gene *Pfkp* is enriched in the MZ (Images were obtained from www.genepaint.org; Visel et al., 2004). **(B)** Treatment of STAT3 inhibitor downregulated expression of the majority of OXPHOS genes. **(C)** Overexpression of STAT3 led to increased OXPHOS genes expression. **(D)** CRISPR-KO of *Stat3* in mESC-derived neural stem cells led to downregulation of OXPHOS genes expression. n=3, Error bars represent mean ± standard deviations.

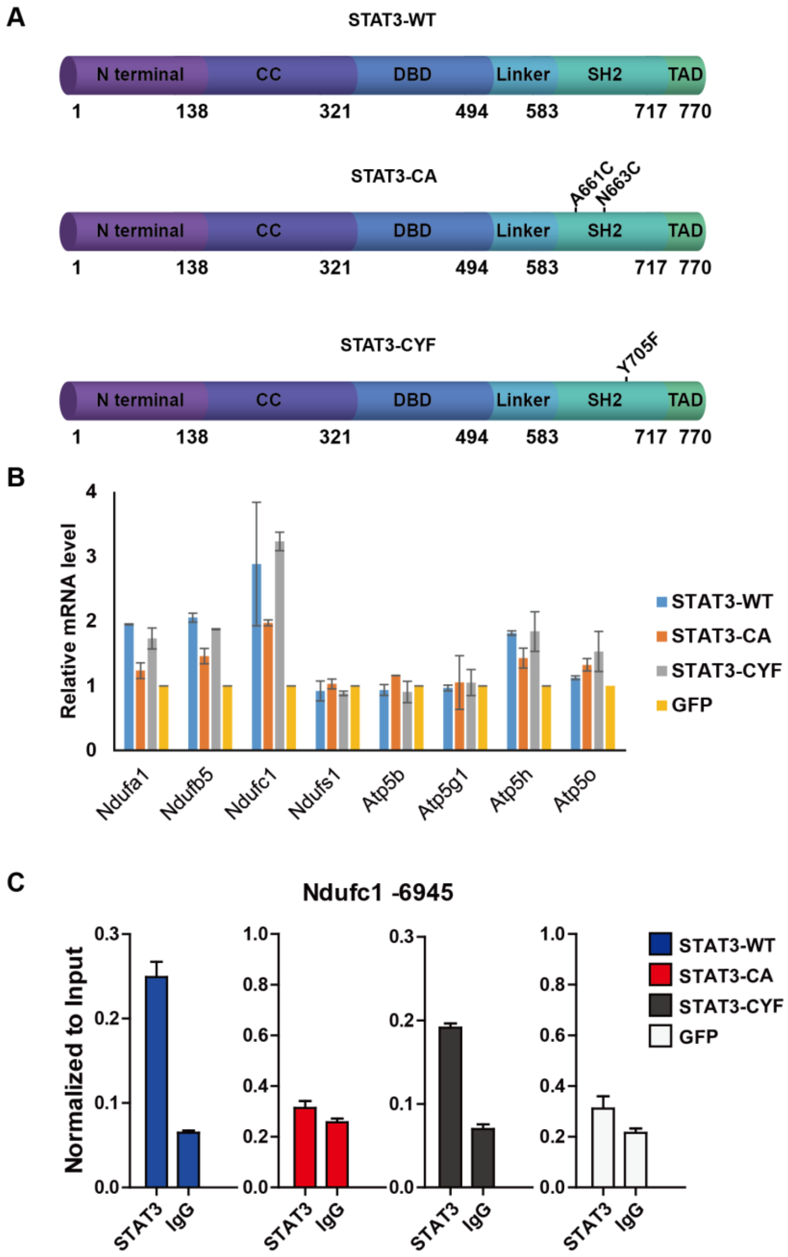

Supplementary Figure S4 Unphosphorylated STAT3 promotes OXPHOS genes expression. **(A)** Schematic view of different STAT3 mutants. STAT3-CA had its Alanine661 and Asparagine663 replaced by cysteine, respectively, to mimic the active form of STAT3. STAT3-CYF had its Tyrosine705 changed to Phenylalanine to inhibit its activation. **(B)** Overexpression of dominant-negative STAT3 (STAT3-CYF) in Neuro2A cell promoted higher OXPHOS genes expression compared to constitutive-active STAT3 (STAT3-CA). **(C)** ChIP-qPCR experiment in Neuro2A cells overexpressed with STAT3-WT, STAT3-CA, STAT3-CYF and GFP. STAT3-CYF had higher affinity to the *Ndufc1* upstream regulatory sequence

compared to STAT3-CA. CC, coiled-coil domain; DBD, DNA binding domain; SH2, Src Homology 2 domain; TAD, transcription activation domain.

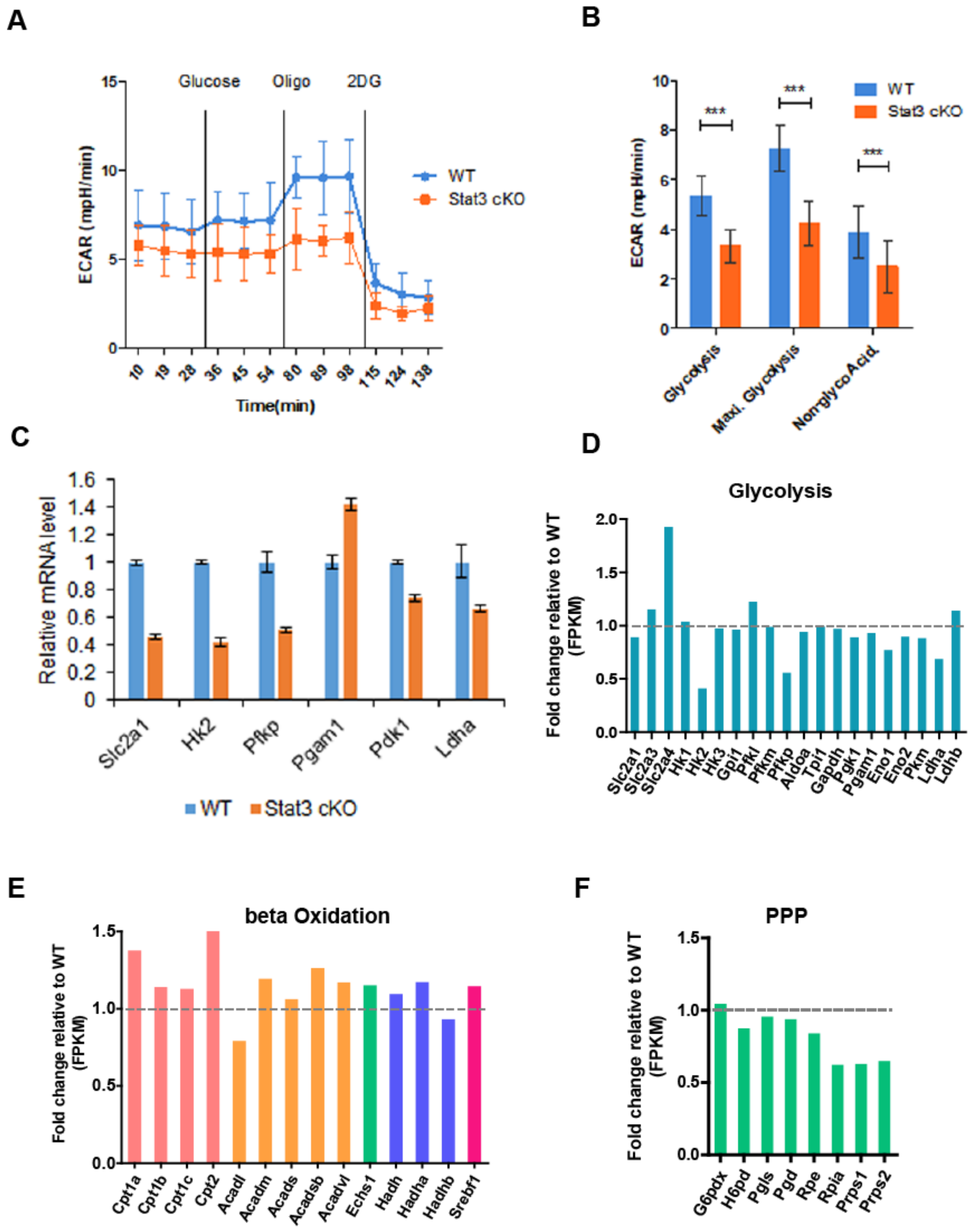

Supplementary Figure S5 Change in other metabolic pathway in *Stat3* cKO neural progenitors. **(A~B)** Seahorse experiment showed ECAR was downregulated in *Stat3* cKO neural progenitors. (n=3, N=4). **(C)** RT-qPCR showed expression of glycolysis pathway genes was downregulated in *Stat3* cKO. **(D~F)** RNA-seq data showed that expression of most of the genes involved in glycolysis and pentose phosphate pathway (PPP) was downregulated in *Stat3* cKO, while that of fatty acid beta-oxidation was upregulated. Error bars represent mean ± standard deviations. *** $p<0.005$

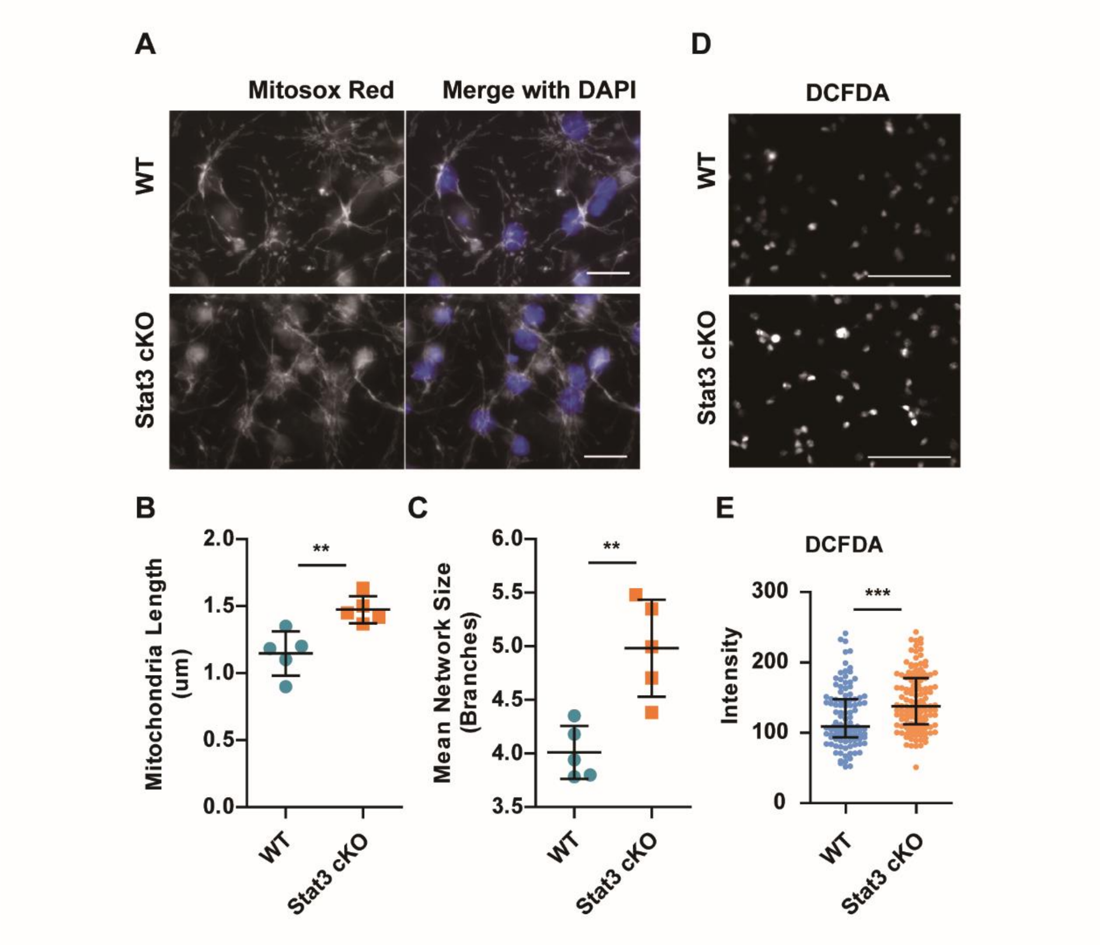

Supplementary Figure S6 STAT3 negatively regulate ROS production and mitochondrial length in neural progenitors. **(A)** Microscopic observation of the Mitosox Red stained neural progenitors. **(B~C)** The length and branch numbers of the MitoSox Red stained mitochondria in neural progenitors were analyzed by ImageJ plugin MiNA. *Stat3* cKO increased the length and the branch number of mitochondria. Each dot in the chart represent the average value in an individual image. Error bars represent mean ± standard deviations. **(D~E)** Microscopic observation showed ROS production level in *Stat3* cKO was higher compared to WT. Dots in **(E)** represent cells from two independent experiments. Error bars represent the median and the interquartile range. ** p<0.01, *** p<0.005, n.s. not significant.

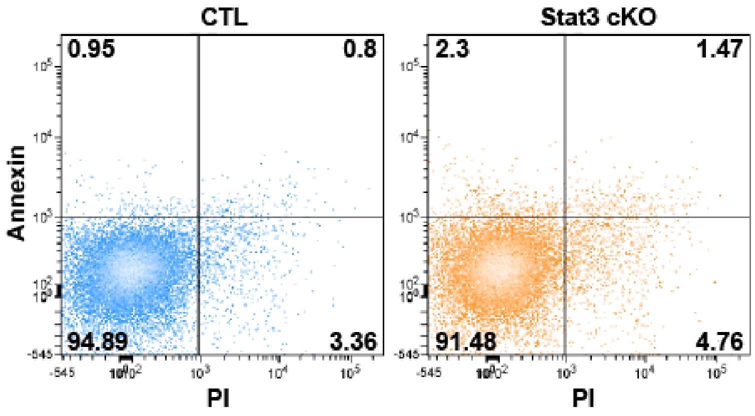

Supplementary Figure S7 *Stat3* cKO lead to slight increase in apoptosis in neural progenitors.

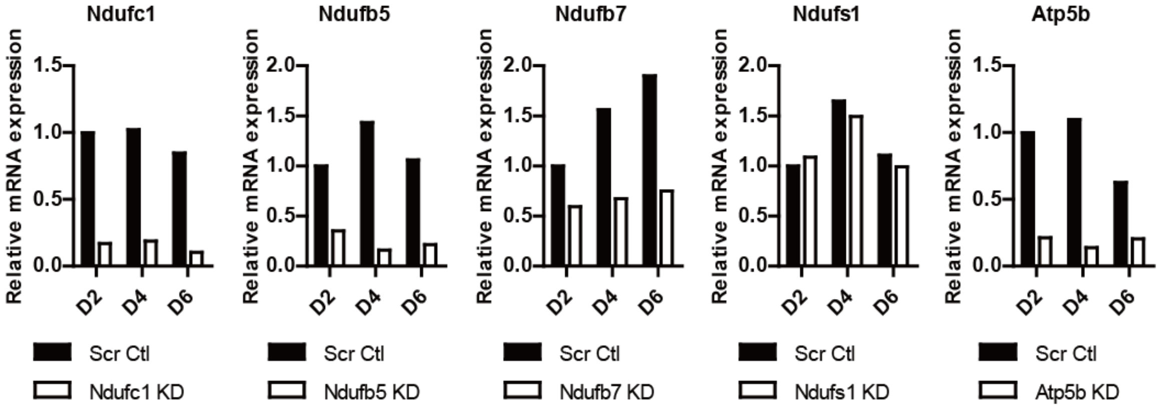

Supplementary Figure S8 Knockdown efficiency validation. RT-qPCR was used to validate the knockdown efficiency at different timepoints. Gene expression was normalized to that of *Gapdh*.

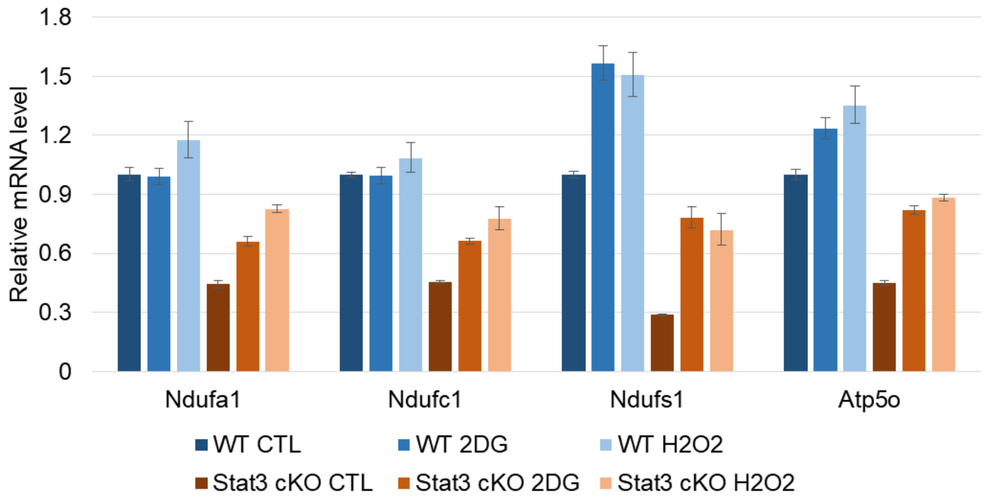

Supplementary Figure S9 Change in the OXPHOS genes expression under drug intervention. RT-qPCR showed that treatment with 4mM 2DG or 10uM H2O2 resulted in the promotion of OXPHOS genes expression. Error bars represent mean ± standard deviations.

Supplementary Table S1 Genotyping primers

Name	Sequence
Stat3-f-F	ATTGGAACCTGGGACCAAGTGG
Stat3-f-R	ACATGTACTTACAGGGTGTGTGC
Nestin-cre-F	CCT TCC TGA AGC AGT AGA GCA
Nestin-cre-R	GCC TTA TTG TGG AAG GAC TG

Supplementary Table S2 SFM recipe

Component (Stock concentration)	100ml	Company	Catolog No.
DMEM/F12	94.3 ml	Invitrogen	1669926
30% D-glucose	2 ml	Sigma	G8270
1M HEPES	0.5 ml	Sigma	H0887
Progesterone (1mg/ml in EtOH)	6.25 ul	Sigma	P8387
Putrescine (10uM)	1 ml	Sigma	P5780
B27	2 ml	Invitrogen	17504-044
ITSS (10mg/ml)	100 ul	Roche,DKSH	100053423
Heparin (25mg/ml)	7.32 ul	Sigma	H0200000

In 100ml SFM, add:

20ul EGF, 500X (sigma: E1257 0.1mg in 1ml PBS/0.1%BSA)

20ul FGF, 500X (sigma: F0291 25ug in 1ml 5mM Tris.HCl PH7.6)

Supplementary Table S3 RT-qPCR Primers

Name	Sequence
mAtp5b_rtqpcr_F	ACCTCGGTGCAGGCTATCTA
mAtp5b_rtqpcr_R	AATAGCCCGGGACAACACAG
mAtp5g1_rtqpcr_F	TTCTCCAGCTCTGATTCGCT
mAtp5g1_rtqpcr_R	GCAGGAAGGCTGCTTAGATG
mAtp5h_rtqpcr_F	TTGACTGGGCTTACTACAGGG
mAtp5h_rtqpcr_R	CAGCTCTTCACATCCTCCTTCT
mAtp5o_rtqpcr_F	TGTTCAGGTCTACGGCATCG
mAtp5o_rtqpcr_R	CCTTCAGGAGTTGCCCTACG
mCyc1_rtqpcr_F	ATCGTTCGAGCTAGGCATGG
mCyc1_rtqpcr_R	GCCGGGAAAGTAAGGGTTGA

mNdufa1_rtqpcr_F	GAGTAACGGTGCGGAGATGT
mNdufa1_rtqpcr_R	AACTCGTTTTTCCTTGCCCC
mNdufb5_rtqpcr_F	ATGACGCTCGCTTCTTGAGG
mNdufb5_rtqpcr_R	GCAAGCTCGGCTTCACCAAT
mNdufb7_rtqpcr_F	ACACTGCAGCAACGTGACTA
mNdufb7_rtqpcr_R	GCGTTTCACGTAATCCAGGTG
mNdufc1_rtqpcr_F	AGCTGCTCTTCAACACGGTC
mNdufc1_rtqpcr_R	TCCACATGAAAACCGAGGCG
mNdufs1_rtqpcr_F	AAAGTGGCCCTCATCGGTAG
mNdufs1_rtqpcr_R	AGCATCCTTTAAGACCTCGCA
mUqcrh_rtqpcr_F	ACTCTGGTTGCGCTTGTTGG
mUqcrh_rtqpcr_R	TCCTCTTCTTCCTCTTTGGGGT
mGapdh_rtqpcr_F	CCCCAGCAAGGACACTGAGCAA
mGapdh_rtqpcr_R	GTGGGTGCAGCGAACTTTATTGATG

Supplementary Table S4 ChIP-qPCR primers

Name	Sequence	Relative Position to TSS
mAtp5b_ChIP_F1	CTGCCACCGCCTAGTAAAGC	-9055
mAtp5b_ChIP_R1	CAGAAAAGGGGCACACATACTT	
mAtp5b_ChIP_F2	GCTTAGCGTATTCTTCCTTTCTAC	-763
mAtp5b_ChIP_R2	GCTCAAGATTCCAGGAGCCA	
mAtp5o_ChIP_F1	CCAAATGAAAAGAGACTACTGGCG	-20
mAtp5o_ChIP_R1	CGGCAGGTACGGACTACAAC	
mCyc1_ChIP_F1	CGAGACCAGGGAGCTCTACTTA	-11290
mCyc1_ChIP_R1	CTCATCCGGGAAGGGTAGTTTG	
mNdufa1_ChIP_F1	GCGCTCGCAGAAATAATGCC	-907
mNdufa1_ChIP_R1	GAGCTTGATGAGGGTCGCTA	
mNdufa1_ChIP_negF	TAGCCGAAAACTTGCCTCCTG	+2634
mNdufa1_ChIP_negR	CTCCAAAACCTTTGCCCGTAA	
mNdufa1_ChIP_F2	AACGTGCACACCTGACGG	-185
mNdufa1_ChIP_R2	CTCCTCCTATCTGATCCGCTG	
mNdufb5_ChIP_F1	GTATGCAGATCACTCACCAAAGC	-3388
mNdufb5_ChIP_R1	AGTCAGGACATTGAGCTAGAACTG	
mNdufb5_ChIP_F2	ATCTGCCGGACAATCTGTGT	-663

mNdufb5_ChIP_R2	CTTCCTCGGACTCGCAGTG	
mNdufb5_ChIP_negF	CATCCACAAAGCGGGAACTTG	+6784
mNdufb5_ChIP_negR	ACAGGTTACGGAGAGTCATGC	
mNdufb7_ChIP_F1	TTCTGTTAAATGTCACCCGTCCT	-5629
mNdufb7_ChIP_R1	ACTTTTACACCTGGTACCCAACA	
mNdufb7_ChIP_F2	CAAAAGAGCTGGGTGTGGTATC	-2713
mNdufb7_ChIP_R2	GAGACAGGATTTCCTCTGTGT	
mNdufb7_ChIP_F3	GTTCATCTCTATGACCCCCGC	-29
mNdufb7_ChIP_R3	CATGGCTACTCTGCCTTACCC	
mNdufb7_ChIP_F4	GAGTCAGAGCTCACCGTCAC	+6483
mNdufb7_ChIP_R4	GGCAGTAACCAAATACTGACTTCTC	
mNdufc1_ChIP_F1	TTCACAAACACACAGATAAACTTCC	-1643
mNdufc1_ChIP_R1	AACCATTATCTTGTAGCTCATCCAA	
mNdufc1_ChIP_F2	TAATTGGGGGTAGAAAGATGGCT	-2229
mNdufc1_ChIP_R2	TCCCTCTCTTCAGACACATCAAA	
mNdufc1_ChIP_F3	TGTCTTCAGACCCACACAAGAA	-1508
mNdufc1_ChIP_R3	AACCAAAACTGTTGGGCTGGA	
mNdufs1_ChIP_F2	GTCTGCGATGGCCAGATTGA	-7091
mNdufs1_ChIP_R2	CTGGGACTCGTTCACTGGAT	
mNdufs1_ChIP_F3	AGGAGTTTTCCGGACGCTCT	-303
mNdufs1_ChIP_R3	GGGGTTTTCAGGTCTCCGAA	
mNdufs1_ChIP_F4	CAAGTTTGTGAGGTTCATGTCTGT	+988
mNdufs1_ChIP_R4	CACCCTGGCTCTAATATCTTGGC	
mNdufs1_ChIP_F5	CCCCAAACTCTTGATGTAGTGC	+1699
mNdufs1_ChIP_R5	GTACTGATGAGGCCAAGCACAG	
mGfap_ChIP_F1	TCCTTTTGTGCCCACGAGT	-1436
mGfap_ChIP_R1	TACAAGCTCCCAGCTCAATAG	
mGfap_ChIP_F2	TGACTCTGGGTACAGTGACCTC	-1
mGfap_ChIP_R2	CTTTATGGAGGAACGGGTTGG	
mGfap_ChIP_F3	ACCCTTTGAAAGAGCATAGCAAA	-2971
mGfap_ChIP_R3	CCAGCGACTGTCCTACAAGC	

Supplementary Table S5 Primers for luciferase reporter generation.

Name	Sequence
mNdufc1_luc_F1_HindIII	cggccaagcttTCACTTCGGATCTGGCCAAC

mNdufc1_luc_R1_XhoI	ctagcctcgagACGTGGTAACTGAAAAGACCG
mNdufc1_mut_F1	ccgcggtcccgataggctccggt
mNdufc1_mut_R1	agcctatcgggaccgcggaggcc
mNdufs1_luc_KpnI_F1	gccggtaccCCAGACTTGCCACAGGTTTCA
mNdufs1_luc_NheI_R1	ctcgctagcTTCAACTTGCCCACTTCAACG
mNdufs1 luc mut F1	GGGCTCGCTCTCGGttATTGCCGAACAGGT
mNdufs1 luc mut R1	ACCTGTTCGGCAATaaCCGAGAGCGAGCCC
mNdufb7_luc_XhoI_F1	ctagc*ctcgag*GTAGTGGAACGAGTGGCACA
mNdufb7_luc_BglII_R1	cctat*agatct*CCTTTCCCTCATCGGCTTGT
mNdufb7 luc mut F1	CTAGATGACCCGGttGACACTGAAGGGGCAGTGCaaCCAGGTGGGAGCG
mNdufb7 luc mut R1	CGCTCCCACCTGGttGCACTGCCCCTTCAGTGTCaaCCGGGTCATCTAG
mAtp5o_luc_F1_HindIII	cggccaagcttGTTTACCAGCAAGGACAGGCA
mAtp5o_luc_R1_XhoI	ctagcctcgagCAGGTTCAAGACGAGTCAACG
mAtp5o_mut_R1	acatttgacagcaagccgagcga
mAtp5o_mut_F1	cggaaaccgactaccgaggaagg

Supplementary Table S6 Primers for shRNA vectors generation

Name	Sequence
Ndufc1_shR_F_BamHI	GATCGAGTTACATGCGAATATGAGTCTCGAGACTCATATTCGCATGTAACTCTTTTTTG
Ndufc1_shR_R_EcoRI	AATTCAAAAAAGAGTTACATGCGAATATGAGTCTCGAGACTCATATTCGCATGTAACTC
Ndufb5_shR_F_BamHI	GATCGCATCCGATATCAAGATGGATCTCGAGATCCATCTTGATATCGGATGCTTTTTG
Ndufb5_shR_R_EcoRI	AATTCAAAAAGCATCCGATATCAAGATGGATCTCGAGATCCATCTTGATATCGGATGC
Ndufb7_shR_F_BamHI	GATCACACAACAAGAGATGATGGATCTCGAGATCCATCATCTCTTGTTGTGTTTTTTG
Ndufb7_shR_R_EcoRI	AATTCAAAAAACACAACAAGAGATGATGGATCTCGAGATCCATCATCTCTTGTTGTGT
Ndufs1_shR_F_BamHI	GATCCCGCTGTTTAATGCTAGAATTCTCGAGAATTCTAGCATTAAACAGCGGTTTTTG
Ndufs1_shR_R_EcoRI	AATTCAAAAACCGCTGTTTAATGCTAGAATTCTCGAGAATTCTAGCATTAAACAGCGG
Atp5b_shR_F_BamHI	GATCGCTAATCAACAATGTCGCCAACTCGAGTTGGCGACATTGTTGATTAGCTTTTTG
Atp5b_shR_R_EcoRI	AATTCAAAAAGCTAATCAACAATGTCGCCAACTCGAGTTGGCGACATTGTTGATTAGC

Scramble_shR_F_BamHI	GATCATCAACCGGATGGACGTTAAA CTCGAGTTTAACGTCCATCCGGTTGATTTTTTG
Scramble_shR_R_EcoRI	AATTCAAAAAATCAACCGGATGGACGTTAAA CTCGAGTTTAACGTCCATCCGGTTGAT
